# No-Reference Quality Assessment of Extended Target Adaptive Optics Images Using Deep Neural Network

**DOI:** 10.3390/s24010001

**Published:** 2023-12-19

**Authors:** Guoqing Gao, Lingxiao Li, Hao Chen, Ning Jiang, Shuqi Li, Qing Bian, Hua Bao, Changhui Rao

**Affiliations:** 1 Key Laboratory of Adaptive Optics, Chinese Academy of Sciences, Chengdu 610209, China; gaoguoqing@ioe.ac.cn (G.G.); chenhao114@mails.ucas.ac.cn (H.C.); jiangning22@mails.ucas.ac.cn (N.J.); bianqing21@mails.ucas.ac.cn (Q.B.); chrao@ioe.ac.cn (C.R.); 2 School of Optoelectronic Science and Engineering, University of Electronic Science and Technology of China, Chengdu 611731, China; 3 Institute of Optics and Electronics, Chinese Academy of Sciences, Chengdu 610209, China; 4 University of Chinese Academy of Sciences, Beijing 101408, China

**Keywords:** image quality assessment, AO images, deep neural network, point spread function

## Abstract

This paper proposes a supervised deep neural network model for accomplishing highly efficient image quality assessment (IQA) for adaptive optics (AO) images. The AO imaging systems based on ground-based telescopes suffer from residual atmospheric turbulence, tracking error, and photoelectric noise, which can lead to varying degrees of image degradation, making image processing challenging. Currently, assessing the quality and selecting frames of AO images depend on either traditional IQA methods or manual evaluation by experienced researchers, neither of which is entirely reliable. The proposed network is trained by leveraging the similarity between the point spread function (PSF) of the degraded image and the Airy spot as its supervised training instead of relying on the features of the degraded image itself as a quality label. This approach is reflective of the relationship between the degradation factors of the AO imaging process and the image quality and does not require the analysis of the image’s specific feature or degradation model. The simulation test data show a Spearman’s rank correlation coefficient (SRCC) of 0.97, and our method was also validated using actual acquired AO images. The experimental results indicate that our method is more accurate in evaluating AO image quality compared to traditional IQA methods.

## 1. Introduction

Image quality assessment (IQA) algorithms aim to reproduce the human perception of image quality. On the one hand, image quality assessment removes poor-quality images and establishes a strong foundation for image post-processing and information mining (e.g., image enhancement, alignment, fusion, and recognition), among others. On the other hand, it also serves as an important metric for evaluating system performance and evaluating image post-processing algorithms.

The IQA can be divided into a subjective assessment and an objective assessment in terms of methods [1]. The subjective assessment assesses the quality of images from human perception, while the objective assessment uses mathematical models to provide quantitative values [2]. IQA models can be classified into three categories, including full-reference (FR), reduced-reference (RR), and no-reference/blind (NR/B) models based on accessibility to a reference [3]. This paper focuses on NR-IQA methods, which can be divided into two categories: hand-crafted feature-based IQA and learning feature-based IQA [4]. Hand-crafted feature-based approaches generally use natural scene statistics (NSS) features to predict image quality scores [5], such as statistical multivariate Gaussian (MVG) models [6], a generalized Gaussian distribution (GGD) model [7], an asymmetric generalized Gaussian distribution (AGGD) [8], multi-channel fused image features [9], and k-nearest neighbor (KNN) based models [10] and so on. So far, NSS-based NR-IQA methods are still being developed. For example, Wang extracted NSS features from a logarithmic field [11] and Dendi used NSS features to assess the quality of a video [12]. However, the ability of the hand-crafted feature-based approach is limited, and it cannot express the high-level semantic information for NR-IQA [1].

With the great success of deep learning in computer vision tasks, some learning-based approaches are also proposed for no-reference/blind image quality assessment. Kang et al. first proposed a convolutional neural network-based NR-IQA method [13]. Hou et al. proposed constructing an image quality assessment model using a fully connected neural network, with NSS features used as the input to train the network [14]. Ma et al. first proposed the Meon model, a multitask-based end-to-end deep convolution network model, to simultaneously learn the distortion type and image quality [15]. Zhu et al. introduced meta-learning into the image quality assessment domain to solve generalization problems and designed the MetaIQA model as a no-reference/blind image quality assessment model [16]. Su et al. separated the IQA procedure into three stages: understanding the content, learning the perception rules, and predicting the quality and proposed a self-adaptive hyper network architecture to blind assess image quality in the wild [17]. You et al. explored the application of the transformer in IQA [18]. Korhonen et al. proposed an RNN-IQA model for assessing high-resolution image quality [19].

Image enhancement, generation, and recovery models have become increasingly popular, thus prompting the development of various methods that can be used to measure their performance. However, most of these methods cater to general image quality assessment and are less reliable when adapting to specific domains, such as adaptive optics (AO) imaging [20]. Consequently, AO is extensively used in ground-based telescopes for high-resolution imaging, laser systems, and ophthalmology. AO systems can correct a significant number of wavefront aberrations [21]. Nevertheless, the imaging quality of AO systems is often adversely affected by various factors, including:

- The wind load, mounting dithering of a telescope, and other factors lead to the overall tilt of the optical axis. The telescope tracking system cannot fully overcome the overall tilt of the optical axis, resulting in motion blur in the AO image;
- The AO system is only capable of partially correcting for wavefront aberrations caused by atmospheric turbulence. As a result, residual higher-order aberrations may lead to diffuse blurring of the image [22].

Mainstreaming algorithms face the challenge of capturing all of the above-mentioned distortions accurately to ensure proper quality prediction. Furthermore, due to the limited availability of datasets and the complexity of the degradation model, no IQA method has been developed specifically for the AO images. Tian et al. proposed entropy measurement as an approach to assess AO images [23]. Guo et al. used the normalized LOG domain [24], and Wen et al. applied the Sobel approach to assess the quality of recovered images after undergoing a similar number of blind convolutions [25]. However, AO images based on ground-based telescopes not only undergo more complex degradation but also differ significantly from natural images in terms of the content:

- Since AO images are grayscale, color features cannot be used to assess them.
- AO images are affected by blurred halos resulting from motion blur and atmospheric turbulence.
- The large black or gray backgrounds present in AO images contain little semantic information.

Furthermore, due to the complex and specific nature of the AO system, there are numerous factors that degrade AO images. Thus, a single image characteristic or distortion model cannot accurately assess AO image quality. The studies mentioned above about AO image assessment are based on image features without considering the special characteristics of AO images and without using the physical information about the imaging process of AO images.

This study proposes an efficient IQA method for extended target AO images, which utilizes a deep neural network to learn the relationship between the degradation factors in the AO imaging process and the image quality. Unlike traditional methods, this method assesses the image quality by extracting the features of the PSF, which can characterize the degradation process of AO images, rather than relying on the image features. This method is more objective than the manual assessment method and is more consistent with the particularity of the AO image than the assessment methods based on image features. Additionally, the AO extended target image dataset created in this paper takes into account the imaging process of AO images.

## 2. Methods

**Figure 1:**
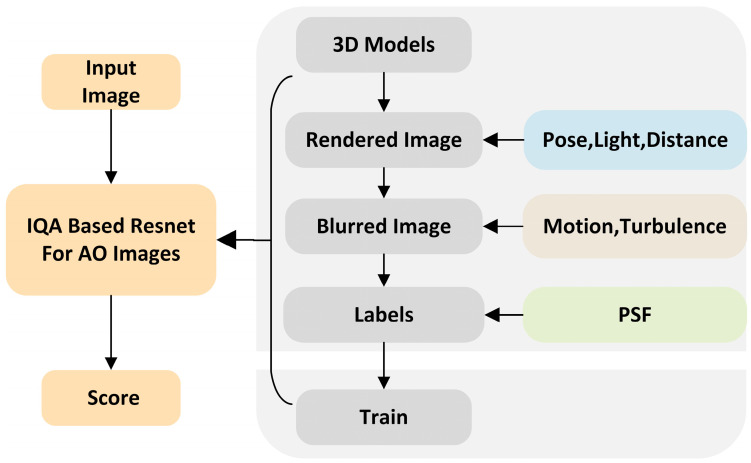
Routine diagram of the proposed method. First, based on the residual aberration of the AO system, the projected 2D images of the expanded target’s 3D model under different conditions are degraded into simulated images. Quality scores are assigned to each frame of the simulated images according to the degradation process’s PSF to construct a dataset for training the IQA network. Finally, real images are inputted to predict their scores.

In this study, diverse 2D rendered images were produced by adjusting the lighting, pose, and distance of various 3D models, which is illustrated in Figure 1. The 2D rendered images were then degraded by accounting for the influence of atmospheric turbulence and motion blur due to system-induced mechanical jitter, resulting in the creation of 400,000 AO simulated images. Next, we generated labels for each of the degraded images based on their PSF. Lastly, the simulated images and their respective quality labels were combined to develop the AO extended target image quality assessment dataset used to train the network in this study.

### 2.1. Degraded AO Images for Extended Target

Firstly, the 3D models of extended objects were constructed according to the collected data from the Internet. Then, we developed a Blender script to obtain different 2D projection images by changing the lighting direction and intensity of the 3D model, the relative angle between the 3D model and the virtual camera, and the distance between the 3D model and the virtual camera.

In this paper, the effects of the residual atmospheric turbulence and the motion blur caused by the system mechanical jitter are considered in the AO degradation procedure [26]:

(1) $$g\left(x,y\right)=f\left(x,y\right)*h\left(x,y\right)*t\left(x,y\right)$$

**Figure 2:**
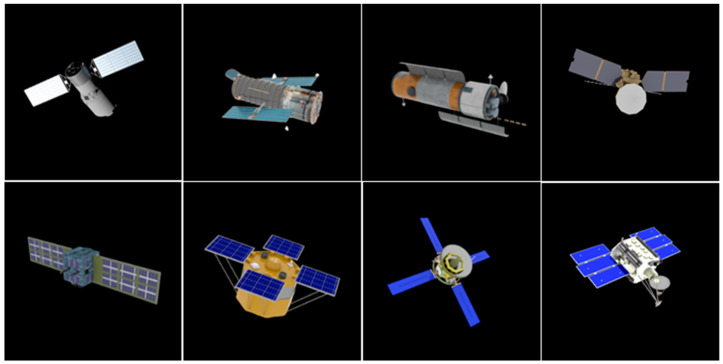
Generated 2D rendered images by changing the lighting direction and intensity of the 3D model, the relative angle between the 3D model and the virtual camera, and the distance between the 3D model and the virtual camera.

**Figure 3:**
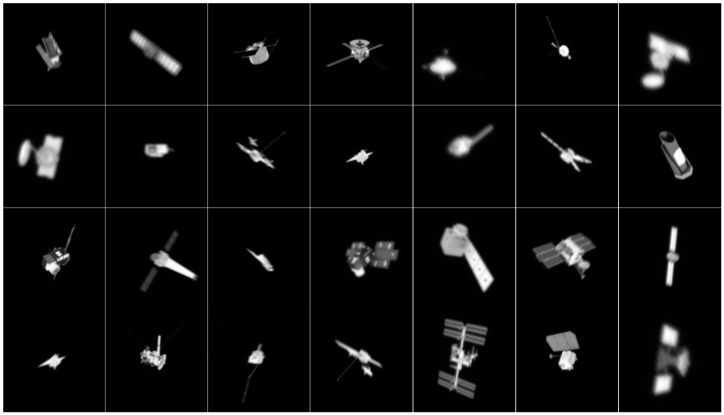
Examples of degraded images by changing the wavefront, RMS of the wavefront, and direction and length of the motion blur.

In Equation (1), $f\left(x,y\right)$ represents the 2D rendered images, such as images shown in Figure 2; $g\left(x,y\right)$ is the degraded image, and examples of $g\left(x,y\right)$ are shown in Figure 3; $h\left(x,y\right)$ is the PSF of atmospheric turbulence; $t\left(x,y\right)$ is the function of motion blur; and * represents convolution. The CCD and stray light in the optical path will introduce background noise, resulting in further degradation of the images, but the impact of such noise can be removed by means of filtering, so the impact of noise is not considered in this paper [27]. A large number of AO degradation simulation images can be generated by changing the parameters of Equation (1). The atmospheric degradation wavefront under the condition of isoplanatic incoherent imaging is completely determined by the wavefront phase, which means that the simulation of the PSF of the atmospheric degradation is equivalent to the numerical simulation for the phase screen of atmospheric turbulence. So, in this paper, we simulated different types of atmospheric turbulence by generating 5 sets of wavefront phases according to the Kolmogorolf spectrum [28] randomly at each value in the RMS. In general, the larger the RMS of the wavefront, the more serious the turbulence. The residual turbulence and motion blur are not too serious because the real AO images are partially corrected by the AO system. In order to better fit the real captured images, we set the RMS between [0.1 and 0.5] after analyzing the images captured by the actual AO system. The motion blur scale was set to between [3, 9] pixels. So, for each rendered image, we randomly selected 5 RMS values of the wavefront at [0.1, 0.5]. Similarly, we took 5 numbers as the length of the motion blur in [3, 9] pixels randomly. For each length of motion blur, five directions were randomly selected at [0, 360]. In this study, we combined the random wavefront, RMS of the wavefront, and direction and length of the motion blur to generate 625 degraded images for each rendered image. Figure 3 shows some of the degraded images.

### 2.2. Labels for Simulation AO Images

The PSF, defined as the response of an imaging system to a point light source, is an important indicator to measure the quality of an imaging system. The PSF is a comprehensive representation of the residual aberrations in optical systems.

**Figure 4:**
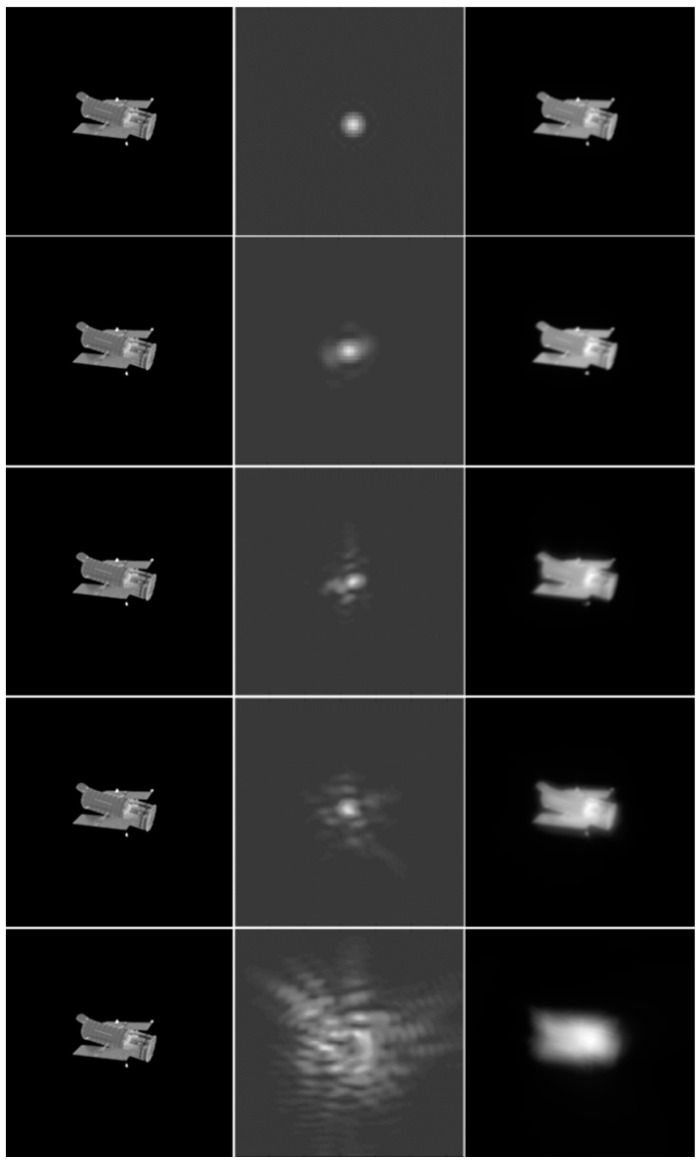
Rendered images are shown in left column, PSFs are shown in middle column, and degraded images are shown in right column. The middle image in the first row is an Airy spot, and the PSFs become more and more diffused from top to bottom. Consequently, the quality of the corresponding degraded images becomes worse and worse.

If the distortion effects of atmospheric turbulence and imaging systems are not considered, an ideal point source of light would produce a Fraunhofer diffraction pattern after being imaged by an optical system. Since the apertures of most optical systems are circular in shape, the image appears as a bright central spot surrounded by uniformly decreasing brightness, known as an Airy spot, as shown in the first row of Figure 4. However, due to the distortion of light during transmission, the final image formed will deviate from the shape and energy distribution of the Airy spot, resulting in the PSF. The point spread function is related to both the imaging aberration and the diffraction effect of the optical system, making it an objective and reliable metric for evaluating the imaging quality of an optical system [27]. Based on the aforementioned optical imaging theory, the image formed by an optical system is the convolution of each point in the object image with the corresponding PSF. Therefore, the PSF is an important parameter for assessing the degradation quality of an image. A PSF that is closer to an Airy disk indicates a lower degree of image degradation and better image quality. Figure 4 presents the differences in the obtained images under different PSFs. As depicted in Equation (1), we consider $h\left(x,y\right)*t\left(x,y\right)$ as the PSF of the degraded image. The objective quality score of the image is determined using the normalized correlation coefficient [29] between the PSF and the Airy spot.

(2) $$s=\frac{\frac{1}{mn}\sum_{i=1}^{m}\sum_{j=1}^{n}\left(PSF\left(i,j\right)-\bar{PSF}\right)\left(Airy\left(i,j\right)-\bar{Airy}\right)}{\sqrt{\frac{1}{mn}\sum_{i=1}^{m}\sum_{j=1}^{n}{\left(PSF\left(i,j\right)-\bar{PSF}\right)}^{2}}\sqrt{\frac{1}{mn}\sum_{i=1}^{m}\sum_{j=1}^{n}{\left(Airy\left(i,j\right)-\bar{Airy}\right)}^{2}}}$$

In Equation (2), *m* and *n* represent the height and width of the *PSF* and the *Airy*, respectively. $\bar{PSF}$ indicates the average value of the *PSF*. $\bar{Airy}$ indicates the average value of the *Airy*.

To further verify the label generation effectiveness in AO images based on the PSF, the same ideal image was employed for various degrees of atmospheric turbulence and motion blur degradation.

**Figure 5:**
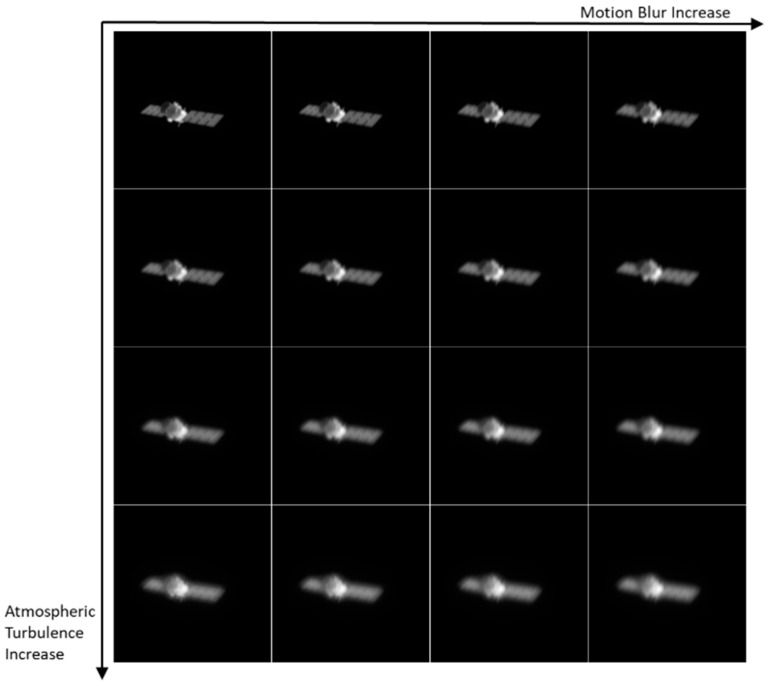
AO simulation image with different degrees of degradation. The motion blur gradually increases from left to right, and the turbulence disturbance gradually increases from top to bottom.

**Figure 6:**
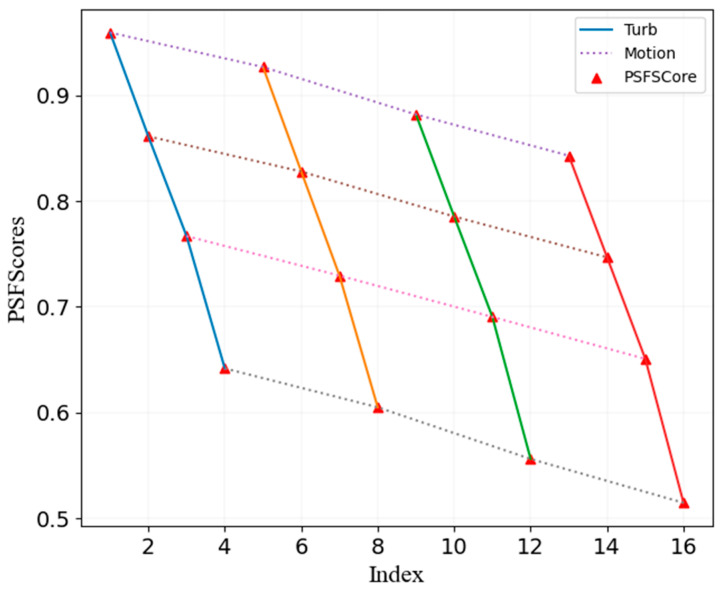
Relationship between labels and degree of degradation. Figure 5 depicts 16 images numbered from left to right and from top to bottom. Equation (2) provides the score presented here.

Figure 5 depicts 16 images numbered from left to right and from top to bottom. Equation (2) provides the score presented in Figure 6, which indicates that the label values decrease from left to right as the motion blur increases. This causes the quality of the images in Figure 5 to decline accordingly. Similarly, the values of the labels decrease from top to bottom as the atmospheric turbulence increases, and the image clarity decreases in Figure 5 accordingly. The increase in the PSF dispersion and distortion results in a more significant decline in the image quality and label value. The proposed PSF-based labeling method accurately reflects the AO image degradation degree and, thus, is useful in generating quality labels.

**Figure 7:**
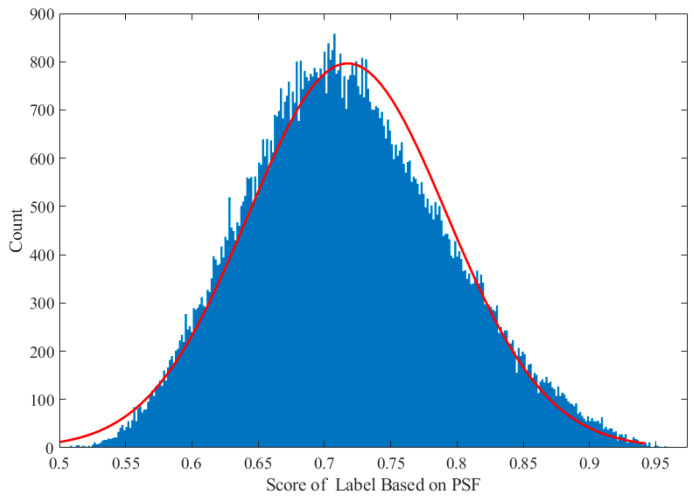
The distribution of quality labels for the degraded AO images approximately conforms to normal distribution.

The distribution of the quality labels for the degraded AO images is shown in Figure 7, which approximately conforms to a normal distribution. The *x*-axis of Figure 7 represents the quality label values for simulated images based on the PSF, and the *y*-axis represents the counts corresponding to those scores. The datasets are divided into training, validation, and testing sets according to 4:1:1 randomly, and they are independently and identically distributed.

### 2.3. Network Model

**Figure 8:**
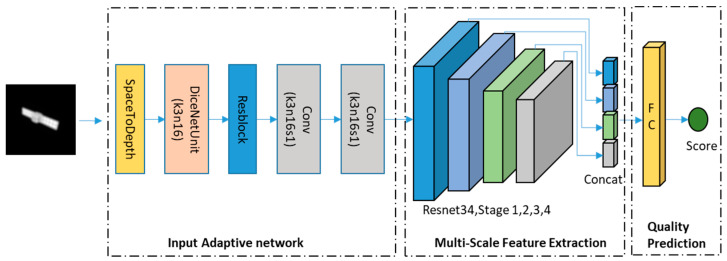
Proposed deep neural network architecture. It comprises an input adaptive module, a multi-scale feature extraction module, and a quality prediction network.

Given that the PSF remains unknown during actual image acquisition and may be challenging to compute [30], reliance on the deep network’s feature representation ability is necessary for analyzing the relationship between the PSF-based image quality score and the input image. The architecture of the proposed AO extended target IQA network is illustrated in Figure 8. It comprises an input adaptive module, a multi-scale feature extraction module, and a quality prediction network.

#### 2.3.1. Input Adaptive Module

To improve the accuracy of the network and reduce the loss caused by downsampling, we pass the image through an input adaptive module before inputting it into the multi-scale feature extraction module. For this paper, the image size was 512 × 512. Downsampling the image to a direct size of 224 × 224 results not only in information loss but also in image quality alteration. Additionally, AO images are single-channel, which means that they are grayscale. As such, we apply the SpaceToDepth [31] and DICEUnit [30] operations to the image to effectively reduce the image quality loss resulting from direct downsampling and to utilize the channel dimension convolution operation to fuse the image features. The SpaceToDepth operation moves the data in the spatial dimensions (width and height) to the depth dimension (channel). The DICEUnit extracts image features from three dimensions: channel, width, and height. The extracted features from the three branches are then fused together as the output of this structure. In this paper, the number of channels was increased through the SpaceToDepth operation, and the features from different channels were fused using the DICEUnit to reduce the loss caused by the image sampling. Then, the image data was passed through a residual block and two 3 × 3 convolutional operations and sent to the multi-scale feature extraction module.

#### 2.3.2. Multi-Scale Feature Extraction Module

To characterize various types of degradation, we employ convolutions to extract features at multiple scales, ranging from local to global [32], which has achieved excellent results in both [17,33]. We concatenate feature maps at various scales as input to the quality prediction network, as outlined in Equation (3) [33]. More specifically, we utilize ResNet50 as the backbone for this task. ResNet50 consists of 4 Residual Blocks. And, we gather feature maps from four different stages of ResNet50. Our network uses ResNet50 as the backbone due to two reasons. Firstly, ResNet50 offers strong feature representation, making remarkable achievements in image processing, and, secondly, it maintains a proper balance between accuracy and speed, achieving high detection performance while consuming limited computing resources.

(3) $$h\left(s\right)=concat(s_{1},\ldots s_{j},\ldots ,s_{n})$$

#### 2.3.3. Quality Prediction Network

To map learned multi-scale image features to a quality score, we use a small and simple quality prediction network, which consists of a fully connected layer. We deploy a sigmoid function as the activation function and use the mean square error as the loss function. Specifically, after the data output from the multi-scale feature extraction module is subjected to average pooling, it goes through FC (2048) and obtains the final predicted quality score.

We implemented the algorithm proposed in this paper using the PyTorch deep learning framework. The training platform was a single NVIDIA GeForce GTX 3090 GPU. To facilitate training, the network was assigned a small initial learning rate of 1 × 10^−3^, which was altered with each iteration of training based on the initial learning rate. The learning rate was adapted using Equation (4), where T was set to 2 and α was set to 0.8. We used an Adam optimizer with a weight decay of 5 × 10^−4^ to train our model for 500 epochs, with a batch size of 32.

(4) $$\eta =\eta_{0}*\alpha^{\frac{e}{T}}$$

### 2.4. Metrics

The prediction performances were evaluated based on the Spearman rank order correlation coefficient (SRCC), the Pearson correlation coefficient (PLCC), and the root-mean-square error (RMSE) between the predicted and ground truth image quality scores. We chose these evaluation measures as they are commonly used to evaluate image quality assessment of natural images [34].

The PLCC describes the linear correlation between two sets of data with values ranging from −1 to 1. When the PLCC value equals zero, the two sets of data are not correlated. When the PLCC value equals 1 or −1, this indicates a complete positive correlation or a negative correlation between two sets of data.

(5) $$PLCC=\frac{\sum_{i=1}^{N}\left(x_{i}-\bar{x}\right)\left(y_{i}-\bar{y}\right)}{\sqrt{\sum_{i=1}^{N}{\left(x_{i}-\bar{x}\right)}^{2}\sum_{i=1}^{N}{\left(y_{i}-\bar{y}\right)}^{2}}}$$

The SRCC analyses the linear correlation by computing the rank size of two sets of variables, without requiring the distribution of the original variables. The range of values for the SRCC is from 0 to 1 inclusive, with higher values indicating a stronger correlation between the two sets of data.

(6) $$SRCC=1-\frac{6\sum_{i=1}^{N}d_{i}^{2}}{N\left(N^{2}-1\right)}$$

The RMSE indicates how dissimilar the predicted and label values are from each other.

(7) $$RMSE=\sqrt{\frac{\sum_{i=1}^{N}{\left(x_{i}-y_{i}\right)}^{2}}{N}}$$

In Equations (5)–(7) [34], N represents the number of images, $x_{i}$ represents the predicted score of the IQA algorithm for the $i^{th}$ image, $y_{i}$ represents the label value for the $i^{th}$ image, and $d_{i}$ represents the difference between the rank of $x_{i}$ and the rank of $y_{i}$.

## 3. Results

To assess the viability of our proposed method, three classical NR-IQA methods, i.e., Tenengrad [25], LOG [24], and HyperIQA [17], were employed to compare our method’s effectiveness on both simulated and real data. The Tenengrad and LOG IQA methods are based on image NSS features and have been utilized in AO image assessment. The Tenengrad method extracts gradient values in horizontal and vertical directions by using the Sobel operator and utilizes the sum of squares as an assessment function. The LOG method normalizes the input image into a LOG domain. HyperIQA is a learning-based IQA method that has exhibited superior results on real distorted images. We used the default configurations as provided by the authors to compare the methods.

We conducted tests separately on simulation images and real captured images. The parameters used for the degradation algorithm in the simulation images were consistent with those of the real AO system. The specific parameters are shown below. The parameters of the actual AO imaging system are as follows: (1) Focal length: 840 mm (for small field of view); (2) Wavelength: using 1.0–1.3 μm filter, with the center wavelength of 1.15 μm; (3) Spectral range: 300 nm; (4) CCD pixel size: 15 μm; (5) Aperture diameter: 36 mm; (6) System diffraction limit half-width: 1.8 pix. The abovementioned parameters are consistent with the parameters of the algorithm used to attain the degraded simulation images.

### 3.1. Simulation Images

**Figure 9:**
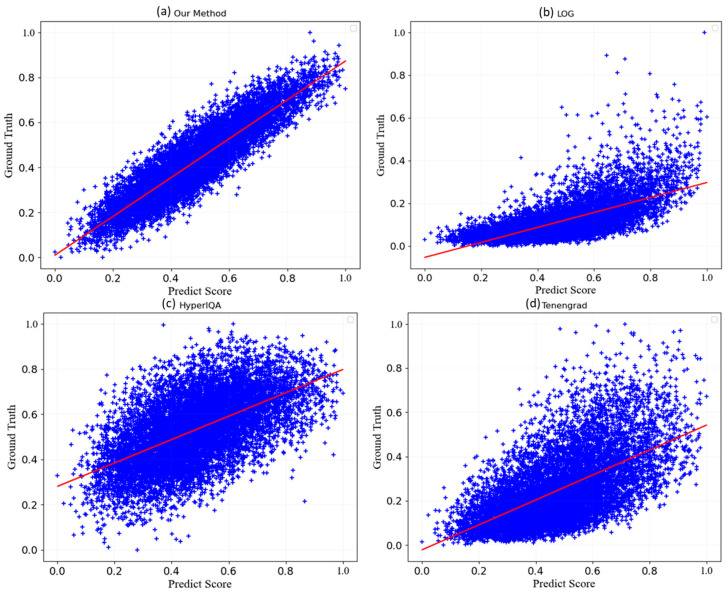
Different IQA methods against labels: (**a**) Our method (Upper left), (**b**) LOG (Upper right), (**c**) HyperIQA (Lower left), and (**d**) Tenengrad (Lower right). The normalized score value of the abovementioned method is plotted on the abscissa axis, where higher score values indicate better image quality based on the method.

Figure 9 demonstrates that the label value is the ordinate of all the sub-images, and the label value increases with the image’s enhanced quality. Meanwhile, the normalized score value of the abovementioned method is plotted on the abscissa axis, where higher score values indicate better image quality based on the method. The imaging results of LOG and Tenengrad are pictured in Figure 9, respectively. Though these two methods generally reflect the observable trend of image quality changes, they produce numerous discrete points and, hence, inaccurate results. Additionally, they generate low label values with high scores, impairing their deliverance of refined results. The imagery produced by HyperIQA, as represented in Figure 9, is not appropriate for AO images due to a more extensive presence of discrete points. In contrast, the outcome of our method, as depicted in Figure 9, establishes a proportional relationship between the trained model’s score value and the resulting label value and, therefore, produces fewer discrete points. As such, this confirms the effectiveness of our method in the simulation images.

It can be observed from Table 1 that the IQA for the AO images presented in this study outperforms other methods on the testing dataset.

### 3.2. Real Images

**Figure 10:**
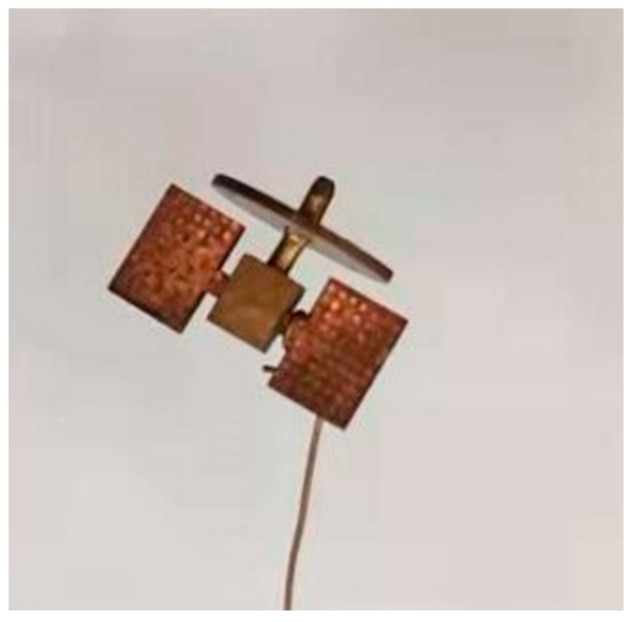
Image of real target.

To test the accuracy and stability of the methods, we built an AO platform in the laboratory, and the specific parameters of the platform are described in Section 3. We used the platform we built to collect a set of images. Specifically, a real target of Figure 10 was placed at the focal position of the AO platform and illuminated by an external light source. We used the atmospheric turbulence simulator, which generated turbulence by heating the air to simulate atmospheric turbulence, and obtained AO images with varying quality.

**Figure 11:**
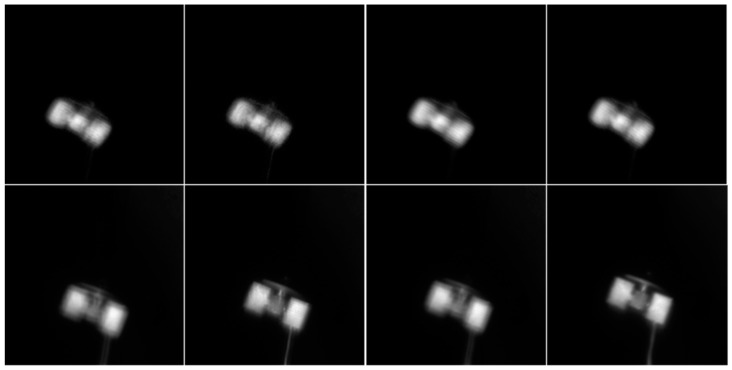
Degraded images (**up**) against real images (**down**). The images generated by this simulation are very similar to the actual images, both visually and physically.

In order to compare the simulated degraded images presented in this study with the images acquired by the AO system, we compared the simulated degraded images according to Equation (1) in Figure 10 to the images obtained by the AO system. As shown in Figure 11, the images generated by this simulation are very similar to the actual images, both visually and physically.

**Figure 12:**
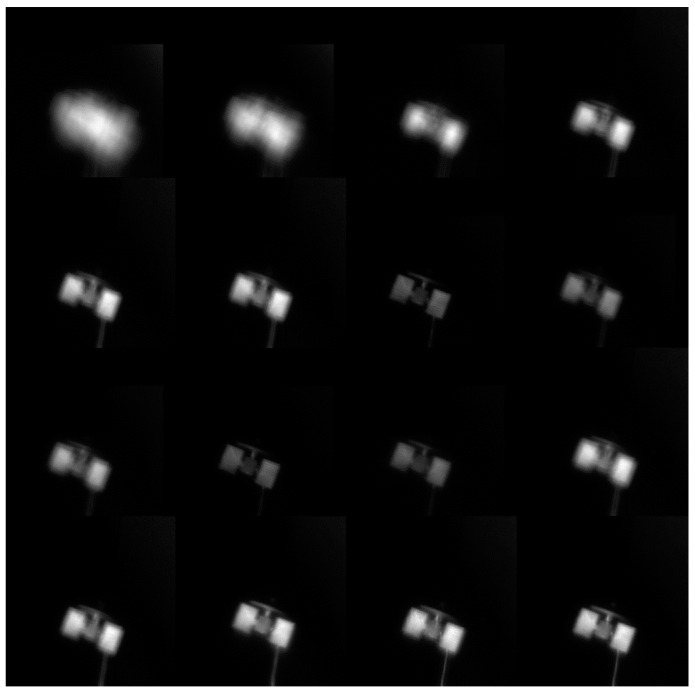
Real AO images from weak to strong. We controlled the degree of correction for the AO system by changing the correction voltage applied by the corrector. Different levels of correction resulted in varying image quality.

We controlled the degree of correction for the AO system by changing the correction voltage applied by the corrector. The recorded RMS value of the wavefront served as the metric for evaluating the system correction effectiveness. Finally, we randomly selected 16 images from the acquired images and sorted them according to the degree of correction from the weakest to the strongest. In Figure 12, from left to right and then down, the RMS value increases.

We compared the proposed method and the IQA methods of Tenengrad, LOG, and HyperIQA, respectively, against the degree of correction in the AO system.

**Figure 13:**
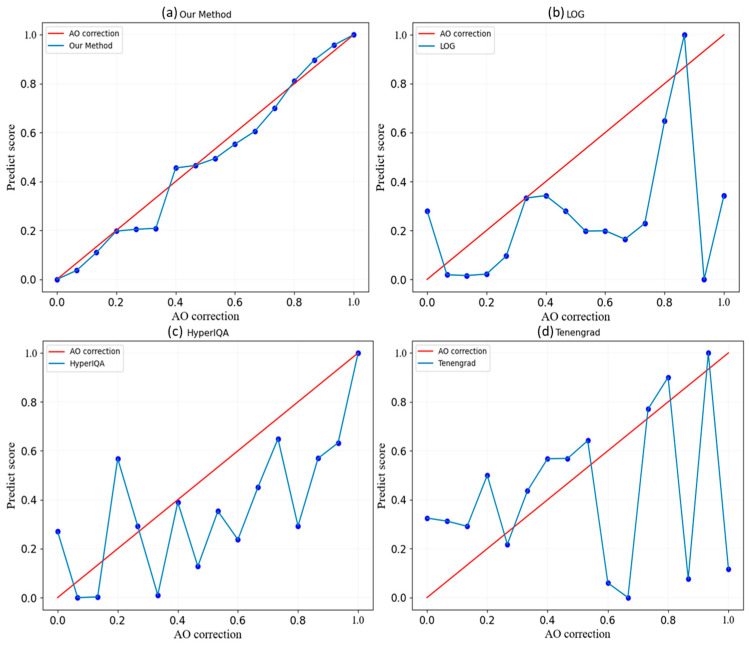
Different methods against the AO correction: (**a**) Our method (Upper left), (**b**) LOG (Upper right), (**c**) HyperIQA (Lower left), and (**d**) Tenengrad (Lower right).

The proposed method in this paper is designed for AO images, and its results match the correction degree of the AO system. The Tenengrad, LOG, and HyperIQA methods shown in Figure 13 match the degree of correction of the AO system only in the local area.

## 4. Discussion

From the tests of the simulated and real images, we can see that although the Tenengrad and LOG methods based on the NSS feature of the image have been applied in the AO image quality assessment, their performance is poor. These two methods only extract one type of image feature as an evaluation criterion. However, the imaging process of AO images is complicated, with multiple factors that degrade the image quality. A single image feature cannot well reflect the image quality and the system’s correction state. The HyperIQA, which performs well in the field of natural distortion images, cannot be well adapted to AO images. Although HyperIQA utilizes the powerful learning ability of neural networks, the quality evaluation of natural images is sensitive not only to degradation models but also to the image content. AO images have a single content, and the target shape is irrelevant to the imaging quality and the system correction capability. The method proposed in this paper maps the multi-scale features of the image onto the PSF reflecting the imaging process, which can better reflect the imaging quality of the AO system. Meanwhile, we implemented the algorithm proposed in a computer with an Intel Core I9-10900X CPU, 32 GB RAM, and NVIDIA GeForce GTX 3090. The computation time for 10,000 AO images of our method is 165 s, and the IQA speed is approximately 61FPS, which meets the online IQA requirements for AO extended target images.

## 5. Conclusions

This paper introduces an IQA network designed to establish the relationship between degradation factors in the AO imaging process and the image quality of AO extended target images. The AO extended target image dataset, specifically created for this research, takes into account the critical factors affecting image quality, such as turbulence and jitter. The results of this study indicate that the PSF of the degraded image serves as a superior quality metric for AO images in comparison to image features. The dataset construction process uncovers the association between the PSF, the AO system’s correction capability, and the image quality. Nonetheless, given that the PSF remains unknown during actual image acquisition and may be challenging to compute, reliance on the deep network’s feature representation ability is necessary for analyzing the relationship between the PSF-based image quality score and the input image. The SRCC on the test data was 0.97, and our method was validated on actual acquired AO images as well. Furthermore, our approach addresses the limitations of AO IQA and image selection, offering an efficient solution for preserving system resources. Additionally, the method supplies a solid foundation for image post-processing and evaluation criteria. The AO image assessment results can aid in optimizing system parameters, thereby enhancing system performance. In future work, we plan to improve the network structure and expand the dataset, allowing the application of the method proposed in this study to be extended to assessing image quality affected by atmospheric turbulence.

## Figures and Tables

**Table 1:** Performance comparison for AO image different IQA methods.

Methods	SRCC	PLCC	RMSE
Tenengrad	0.673	0.656	0.075
LOG	0.769	0.729	0.149
HyperIQA	0.656	0.638	0.081
Ours	0.971	0.961	0.008

## Data Availability

The data presented in this study are available upon request from the first author.
